# Trismus and Retropharyngeal Inflammation Revealing Multisystem Inflammatory Syndrome in Children: A Case Report

**DOI:** 10.7759/cureus.111700

**Published:** 2026-06-29

**Authors:** Ikram El Hachmi, Khalil Elouadghiri Fouad, Anane Sara, Aziza Elouali, Maria Rkain, Abdeladim Babakhouya

**Affiliations:** 1 Department of Pediatrics, Mohammed VI University Hospital, Faculty of Medicine and Pharmacy, Mohammed First University, Oujda, MAR

**Keywords:** acute kidney injury, cervical lymphadenopathy, mis-c, myocardial dysfunction, retropharyngeal inflammation, sars-cov-2, shock, trismus

## Abstract

Multisystem inflammatory syndrome in children (MIS-C) is a severe post-infectious hyperinflammatory condition associated with SARS-CoV-2 infection. We report a case of a previously healthy 14-year-old girl who presented with fever, painful cervical lymphadenopathy, progressive trismus, and a diffuse erythematous rash. On admission, she exhibited circulatory shock with severe hypotension, markedly elevated inflammatory markers, acute kidney injury, and severe myocardial dysfunction. Cervical computed tomography revealed retropharyngeal inflammatory edema associated with cervical lymphadenopathy. SARS-CoV-2 serology showed positive IgG and negative IgM antibodies. Based on the clinical presentation, laboratory findings, and multisystem involvement, a diagnosis of MIS-C was established. Treatment with intravenous methylprednisolone pulses followed by oral corticosteroids, aspirin, and supportive therapy resulted in rapid clinical and laboratory improvement, including complete resolution of trismus, recovery of renal function, and normalization of cardiac function. Follow-up echocardiography demonstrated complete recovery of left ventricular systolic function, and the patient remained asymptomatic at three-month follow-up. This case highlights an unusual presentation of MIS-C with trismus and retropharyngeal inflammatory changes. Awareness of this rare manifestation may facilitate early diagnosis and prompt treatment, thereby preventing potentially life-threatening complications.

## Introduction

Multisystem inflammatory syndrome in children (MIS-C) is a recently described, potentially life-threatening hyperinflammatory syndrome that occurs after infection with severe acute respiratory syndrome coronavirus 2 (SARS-CoV-2), commonly referred to as COVID-19 [1,2]. MIS-C has been reported worldwide, with a declining incidence observed across multiple countries. Recent national surveillance data from the United States estimate the current incidence at 0.09-0.14 cases per 1,000,000 person-months, reflecting a decline of approximately 98% since the early phase of the pandemic [3]. Despite its declining incidence, MIS-C remains a clinically significant condition, with mortality estimated at 1-2% [4]. Furthermore, growing evidence highlights the potential for long-term multisystem sequelae following MIS-C [4]. Similar to acute rheumatic fever, MIS-C is considered a post-infectious condition that develops several weeks after SARS-CoV-2 infection, which may be mild or even asymptomatic in children [1].

MIS-C is currently defined by the Centers for Disease Control and Prevention (CDC) and the World Health Organization (WHO) as a severe post-infectious hyperinflammatory syndrome characterized by fever (≥38°C according to the CDC criteria and lasting at least three days according to the WHO criteria), elevated inflammatory markers, multisystem involvement, and evidence of current or previous SARS-CoV-2 infection or exposure, in the absence of an alternative plausible diagnosis [5,6]. The CDC definition further requires illness severity necessitating hospitalization [5,6].

The most commonly reported manifestations of MIS-C involve the cardiovascular and gastrointestinal systems, while mucocutaneous findings such as rash, conjunctivitis, and peripheral edema are also frequently observed [1]. Although uncommon, cervical involvement, including retropharyngeal inflammatory changes, has been reported in association with MIS-C [1,7]. We report a case of a 14-year-old girl who presented with trismus and retropharyngeal inflammatory changes as the initial manifestations of MIS-C, subsequently complicated by circulatory shock, severe myocardial dysfunction, and acute kidney injury.

## Case presentation

A previously healthy 14-year-old girl was admitted to the pediatric emergency department for fever, cervical swelling, and trismus. She had no history of previous symptomatic COVID-19 infection, recent respiratory illness, or known contact with a confirmed COVID-19 case.

Six days before admission, she developed high-grade fever associated with painful left cervical swelling and generalized asthenia. Two days after fever onset, progressive trismus appeared and was followed by the development of a diffuse erythematous rash involving the extremities, including the palms and soles. A 48-h course of outpatient oral amoxicillin-clavulanate was prescribed, but no clinical improvement was observed. Over the following days, the rash extended to the trunk, abdomen, face, and eyelids. Shortly before admission, her clinical condition deteriorated with abdominal pain, oliguria, and increasing fatigue.

At admission, vital signs revealed a temperature of 39°C, blood pressure of 65/33 mmHg, and heart rate of 110 beats/min, consistent with circulatory shock. Physical examination revealed a painful 3-cm left cervical lymphadenopathy, marked trismus limiting mouth opening, and bilateral palpebral erythema without edema. Cutaneous examination showed a diffuse erythematous rash predominantly composed of macular and maculopapular lesions. The lesions were round, well-demarcated, and ranged from 5 mm to 2 cm in diameter. They involved the abdomen, back, and lower extremities, with focal areas of confluence forming larger erythematous plaques (Figures 1A-1E). No vesicles, crusts, excoriations, or desquamation were observed. Oliguria had markedly progressed to anuria.

**Figure 1:**
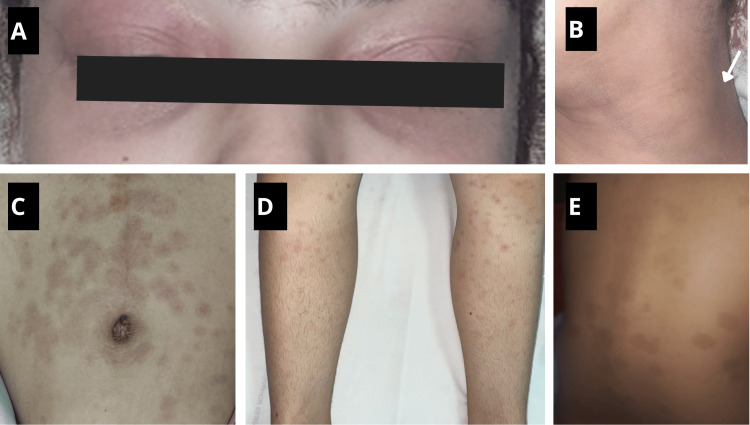
Clinical manifestations at presentation. (A) Bilateral palpebral erythema. (B) Left cervical swelling corresponding to inflammatory cervical lymphadenopathy (arrow). (C) Diffuse erythematous maculopapular rash involving the abdomen. (D) Erythematous maculopapular lesions affecting both lower extremities. (E) Round, well-demarcated erythematous plaques on the back.

Laboratory investigations revealed marked systemic inflammation (Table 1). Complete blood count showed leukocytosis with marked neutrophilia, severe lymphopenia, and a platelet count at the lower limit of the normal range, without thrombocytopenia. Inflammatory markers were markedly elevated, including C-reactive protein, procalcitonin, erythrocyte sedimentation rate, ferritin, and fibrinogen, consistent with a hyperinflammatory state. Cardiac involvement was suggested by a markedly elevated N-terminal pro-B-type natriuretic peptide (NT-proBNP) level. Renal function tests indicated severe acute kidney injury associated with proteinuria. Liver enzymes were also elevated, indicating hepatic involvement. HIV serology was negative. SARS-CoV-2 serology demonstrated positive IgG antibodies and negative IgM, suggesting previous exposure to SARS-CoV-2.

**Table 1:** Evolution of laboratory parameters during hospitalization. GFR: glomerular filtration rate; AST: aspartate aminotransferase; ALT: alanine aminotransferase; NT-proBNP: N-terminal pro-B-type natriuretic peptide

Parameters	Admission	48 h	Discharge	Reference range
White blood cell count (cells/mm³)	22,670	16,600	10,940	4,100-11,000
Neutrophils (cells/mm³)	21,790	14,090	7,840	1,800-8,000
Lymphocytes (cells/mm³)	360	1,870	2,370	1,500-3,500
Hemoglobin (g/dL)	12.0	9.7	10.8	12-16
Platelets (cells/mm³)	155,000	295,000	382,000	150,000-450,000
C-reactive protein (mg/L)	536	140	19.8	<5
Erythrocyte sedimentation rate (mm/h)	119	-	73	<20
Procalcitonin (ng/mL)	241.4	0.51	-	<0.5
Ferritin (ng/mL)	1,372	-	563.9	10-150
Fibrinogen (g/L)	7.9	5.7	4.2	2.0-4.0
Urea (g/L)	1.44	0.60	0.42	0.15-0.45
Creatinine (mg/L)	58.22	14.58	4.33	5-13
Estimated GFR (mL/min/1.73 m²)	17	-	156.38	>90
Proteinuria (mg/kg/day)	25	-	5.5	<4
AST (U/L)	208	100	32	15-40
ALT (U/L)	112	86	35	10-45
NT-proBNP (pg/mL)	>70,000	43,027	1,508	<125

Transthoracic echocardiography revealed severe left ventricular systolic dysfunction with a hypokinetic cardiomyopathy and a left ventricular ejection fraction ranging from 24% to 36%, associated with a mild pericardial effusion (Figures 2A, 2B). No coronary artery abnormalities were identified.

**Figure 2:**
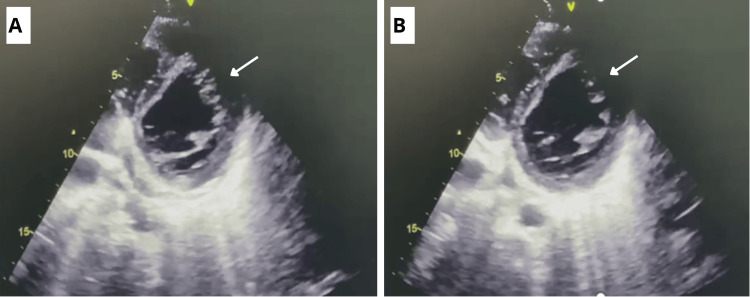
Transthoracic echocardiography demonstrating severe left ventricular systolic dysfunction at admission. (A) Systolic view and (B) diastolic view showing a markedly dilated and hypokinetic left ventricle with reduced ejection fraction. White arrows indicate the left ventricle.

Cervical computed tomography demonstrated left cervical lymphadenopathy associated with retropharyngeal inflammatory edema (Figure 3). These findings explained the patient's trismus and initially raised concern for a deep neck space infection. Abdominal ultrasonography showed no abnormalities.

**Figure 3:**
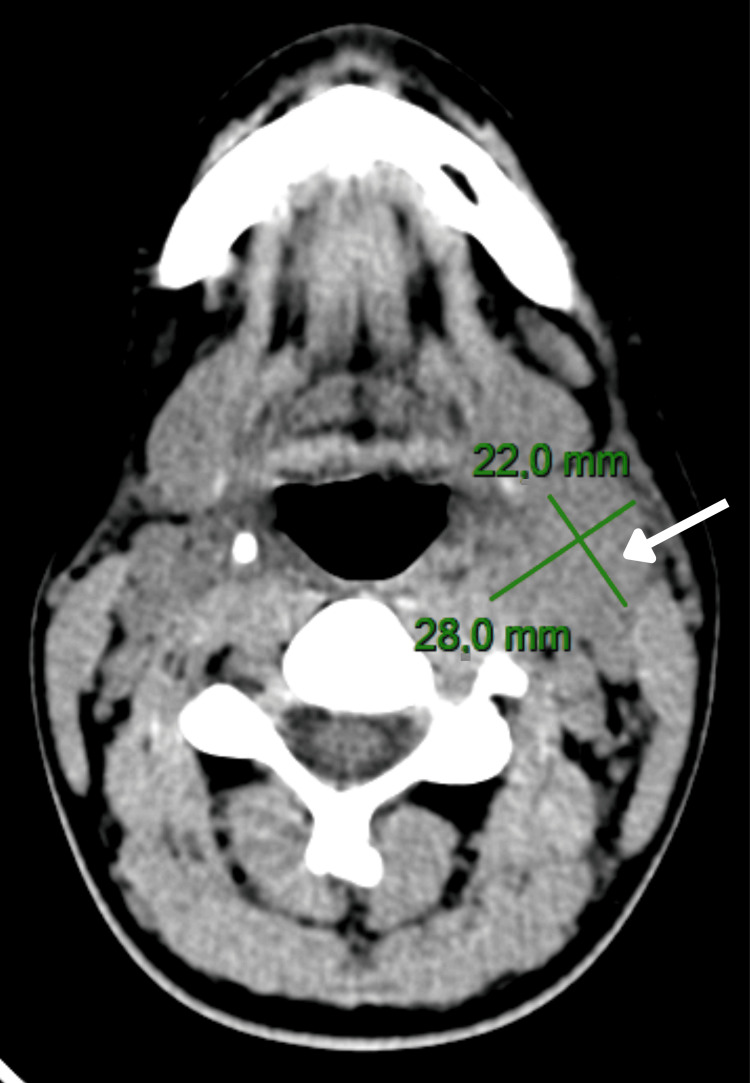
Axial contrast-enhanced cervical CT scan demonstrating left cervical lymphadenopathy. White arrow indicating an enlarged left cervical lymph node measuring 28×22 mm.

The patient fulfilled the World Health Organization (WHO) diagnostic criteria for multisystem inflammatory syndrome in children (MIS-C), including fever persisting for more than three days, markedly elevated inflammatory markers, multisystem involvement (cardiac, renal, hepatic, and mucocutaneous), evidence of prior SARS-CoV-2 infection demonstrated by positive IgG serology, and the absence of an alternative diagnosis after clinical, laboratory, and imaging evaluation [6]. Alternative diagnoses, including bacterial sepsis, toxic shock syndrome, macrophage activation syndrome, Kawasaki disease, and retropharyngeal abscess, were considered but were not supported by the overall clinical, laboratory, and imaging findings.

Treatment with intravenous methylprednisolone pulses (1 g/1.73 m²/day for three consecutive days) was initiated, followed by oral corticosteroid therapy. High-dose aspirin (50 mg/kg/day) was administered during the acute phase. Supportive management included cautious fluid resuscitation, furosemide, and digoxin because of significant myocardial dysfunction. Intravenous immunoglobulin was not administered because of the rapid clinical response to corticosteroid therapy and local availability constraints.

Clinical improvement was rapid after treatment initiation. Fever resolved within 48 h, and the skin rash progressively disappeared. Trismus markedly improved, with complete recovery of oral opening. Urine output normalized within 72 h, and hemodynamic parameters stabilized. Serial laboratory assessments demonstrated a marked decline in inflammatory markers, NT-proBNP levels, liver enzymes, and serum creatinine (Table 1). During recovery, characteristic mucocutaneous desquamation involving the eyelids and palmar aspects of the fingers was observed (Figures 4A, 4B).

**Figure 4:**
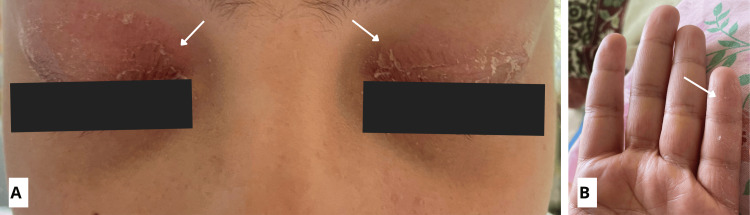
Convalescent mucocutaneous manifestations of MIS-C. (A) Bilateral palpebral desquamation (white arrows) observed during recovery. (B) Digital desquamation (white arrow) during the convalescent phase. MIS-C: multisystem inflammatory syndrome in children

At discharge, oral corticosteroid tapering was continued for 15 days, while low-dose aspirin was maintained for three months. A follow-up echocardiogram performed 15 days after discharge demonstrated recovery of left ventricular systolic function, with an ejection fraction of 66% and no evidence of coronary artery abnormalities (Figures 5A, 5B). Mild residual left ventricular dilation persisted. At the three-month follow-up, the patient remained asymptomatic, and repeat echocardiography was completely normal.

**Figure 5:**
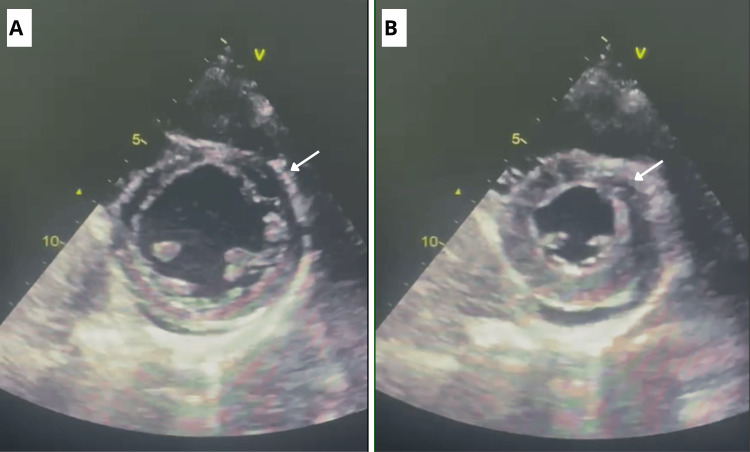
Follow-up echocardiographic assessment. (A) End-diastolic parasternal short-axis view and (B) end-systolic parasternal short-axis view. The examination demonstrates recovery of left ventricular systolic function with a left ventricular ejection fraction of 66%. White arrows indicate the left ventricle.

## Discussion

Since the emergence of the SARS-CoV-2 pandemic, multisystem inflammatory syndrome in children (MIS-C) has been recognized as a severe post-infectious hyperinflammatory condition affecting children and adolescents. While cardiovascular, gastrointestinal, and mucocutaneous manifestations are commonly reported, cervical presentations remain relatively rare [1].

Our patient presented with painful cervical lymphadenopathy, progressive trismus, and retropharyngeal inflammatory edema. Cervical lymphadenopathy is a common pediatric finding, usually reflecting an underlying infectious or inflammatory process. It is frequently encountered in deep neck infections and has also been described in systemic inflammatory disorders such as Kawasaki disease [1]. Retropharyngeal edema, initially described in Kawasaki disease, has subsequently been identified in MIS-C, where it may mimic a retropharyngeal abscess [7,8].

In our patient, cervical imaging revealed retropharyngeal inflammatory edema associated with painful cervical lymphadenopathy and trismus. The marked clinical improvement observed after initiation of immunomodulatory therapy, together with the absence of surgical intervention, suggests that systemic inflammation may have contributed substantially to these cervical manifestations. The pathogenesis of cervical involvement has not been fully elucidated but may be associated with lymphatic dysfunction and activation of the extensive cervical lymphatic network in the setting of severe systemic inflammation [1,7]. Recognition of this presentation is important because it may mimic deep neck infections and delay the diagnosis of MIS-C.

Cardiovascular involvement was prominent in our patient, manifesting as circulatory shock, severe left ventricular systolic dysfunction, and markedly elevated N-terminal pro-B-type natriuretic peptide (NT-proBNP) levels. This finding is consistent with previous reports identifying cardiovascular dysfunction as one of the most severe manifestations of MIS-C, frequently associated with hypotension and echocardiographic abnormalities [9]. Following corticosteroid therapy and supportive cardiac management, our patient experienced rapid hemodynamic stabilization, progressive normalization of NT-proBNP levels, and recovery of left ventricular systolic function.

Acute kidney injury (AKI) was another notable feature of our case, with severe renal impairment at presentation. Although less frequently reported than cardiovascular involvement, AKI has been reported in approximately 2-8% of children with MIS-C in pediatric studies from the United States [2,10]. Its pathogenesis appears to be multifactorial, involving an interplay of systemic hyperinflammation, cytokine release, macrophage activation, microvascular injury, and circulatory impairment. Furthermore, the delayed onset of MIS-C after SARS-CoV-2 infection, together with the marked inflammatory response characteristic of the syndrome, supports the role of post-infectious immune dysregulation in the development of organ injury [2,11]. In our patient, renal function progressively normalized following immunomodulatory therapy and recovery from circulatory shock.

The positive SARS-CoV-2 IgG serology and negative IgM findings further support the post-infectious nature of MIS-C. Previous studies have shown that the syndrome typically develops several weeks after SARS-CoV-2 infection, often following an asymptomatic or mildly symptomatic course. This temporal pattern may explain the absence of recent COVID-19 symptoms in our patient [12].

Beyond the acute phase, emerging evidence suggests that MIS-C may be associated with long-term multisystem morbidity extending well beyond hospital discharge, highlighting the importance of structured follow-up even in patients who achieve complete clinical and echocardiographic recovery [4]. In our patient, follow-up echocardiography at three months demonstrated normalization of left ventricular function and resolution of the mild residual dilation observed during the convalescent phase. Although these findings are reassuring, recent long-term follow-up studies suggest that delayed or subclinical sequelae may occur in a subset of children with MIS-C, supporting the need for continued surveillance [4].

This case illustrates an uncommon presentation of MIS-C characterized by trismus and retropharyngeal inflammatory changes initially suggestive of a deep neck infection. Awareness of such atypical manifestations may facilitate earlier recognition of MIS-C and timely initiation of appropriate treatment, particularly in children presenting with cervical inflammation associated with systemic involvement.

## Conclusions

MIS-C may present with predominant cervical manifestations, including cervical lymphadenopathy, trismus, and retropharyngeal inflammatory changes, potentially mimicking deep neck infections and delaying diagnosis. Clinicians should consider MIS-C in any febrile child with unexplained cervical inflammation, especially when accompanied by shock, multisystem involvement, or laboratory evidence of hyperinflammation. Early recognition of this atypical presentation and prompt initiation of immunomodulatory therapy are crucial to improve outcomes and prevent potentially life-threatening complications, including cardiovascular dysfunction and multiorgan failure.
